# A triple-step controller with linear active disturbance rejection control for a lower limb rehabilitation robot

**DOI:** 10.3389/fnbot.2022.1053360

**Published:** 2022-11-24

**Authors:** Huanfeng Peng, Jie Zhou, Rong Song

**Affiliations:** ^1^The Key Laboratory of Sensing Technology and Biomedical Instrument of Guangdong Province, School of Biomedical Engineering, Sun Yat-sen University, Guangzhou, China; ^2^School of Biomedical Engineering, Shenzhen Campus of Sun Yat-sen University, Shenzhen, China

**Keywords:** lower limb rehabilitation robot, triple-step method, linear active disturbance rejection control, dynamic uncertainties, trajectory tracking

## Abstract

Lower limb rehabilitation robots (LLRRs) have shown promising potential in assisting hemiplegic patients to recover their motor function. During LLRR-aided rehabilitation, the dynamic uncertainties due to human-robot coupling, model uncertainties, and external disturbances, make it challenging to achieve high accuracy and robustness in trajectory tracking. In this study, we design a triple-step controller with linear active disturbance rejection control (TSC-LADRC) for a LLRR, including the steady-state control, feedforward control, and feedback control. The steady-state control and feedforward control are developed to compensate for the gravity and incorporate the reference dynamics information, respectively. Based on the linear active disturbance rejection control, the feedback control is designed to enhance the control performance under dynamic uncertainties. Numerical simulations and experiments are conducted to validate the effectiveness of TSC-LADRC. The results of simulations illustrate that the tracking errors under TSC-LADRC are obviously smaller than those under the triple-step controller without LADRC (TSC), especially with the change of external loads. Moreover, the experiment results of six healthy subjects reveal that the proposed method achieves higher accuracy and lower energy consumption than TSC. Therefore, TSC-LADRC has the potential to assist hemiplegic patients in rehabilitation training.

## Introduction

Globally, stroke is a major threat to human health, and post-stroke care has brought a substantial economic burden to society (Johnson et al., 2019). Due to brain injury, stroke often leads to lower limb dysfunction, which greatly reduces patients' quality of life (Hobbs and Artemiadis, 2020). Therefore, rehabilitation training is needed to help these patients recover their motor function or reduce the risk of several medical consequences secondary to paralysis, such as muscle atrophy and obesity (Chen et al., 2016). In traditional rehabilitation training, the physiotherapist manually guides the patients with impaired limbs to perform repetitive movement training, which is labor-intensive and difficult to quantitatively assess the level of recovery (Akdogan and Adli, 2011). In order to reduce the workload of physiotherapists and enhance the rehabilitation effect, many studies have been conducted on lower limb rehabilitation robots (LLRRs), such as LOPES (Veneman et al., 2007), HAL (Sankai, 2007), and Lokomat (Riener et al., 2005).

Controllers are the critical factor determining the effectiveness of LLRR-aided rehabilitation (Hussain et al., 2013). Among the present research works, most controllers are designed to assist dysfunctional lower limbs in tracking a predefined trajectory (Li et al., 2021). As a model-free controller with a simple and generic control structure, the proportional-integral-derivative (PID) controller has been widely applied to LLRRs (Wu et al., 2015; Zhang et al., 2016; Al-Waeli et al., 2021). However, due to the underutilization of model information, the robotic systems based on the PID controller show poor robustness to external disturbances. Therefore, model-based controllers are proposed to strengthen the anti-disturbance ability of LLRRs. Shen et al. (2020) combined the kinematics and friction models with adaptive robust position control to improve the tracking performance of LLRR under a complex interaction environment. Hernández et al. (2020) designed a non-singular fast terminal sliding mode control for a powered four-degree-of-freedom LLRR, showing strong robustness to external disturbances. Based on a unilateral human-robot dynamical model, a robust controller was designed to drive a LLRR to follow a pre-specified trajectory (Qin et al., 2020). In fact, the LLRR system is characterized by non-linearity, hence the calculation and deduced process of the designed controllers is complicated. Inspired by the triple-step method (Gao et al., 2014; Zhou et al., 2019) proposed a triple-step non-linear controller for LLRR to guarantee control accuracy under different levels of interaction torque. The triple-step method simplified the complicated design of a non-linear controller as a triple-step design process, including the design of steady-state control, feedforward control, and feedback control. On this basis, the structure of the deduced controller was concise.

Dynamic uncertainties of the LLRR system are the main issue that should be considered in controller design (Li et al., 2021). In the LLRR-aided rehabilitation training, the dynamic uncertainties such as human-robot coupling, model uncertainties, and external disturbances, significantly affect the tracking performance. Owing to the non-linear mapping capability, Zhang et al. (2020) combined a radial basis functions neural network (RBFNN) with a sliding mode controller to approach and compensate for the model uncertainties and external disturbances. Besides, Huang et al. (2022) integrated a disturbance observer (DO) into the controller design to compensate for dynamic uncertainties. Khamar et al. (2021) used a non-linear DO in the backstepping sliding controller to assess the wearer's muscle effort and the uncertainties in modeling. Although the control performance of LLRR can be improved by the RBFNN and DOs, the parameters they introduce are difficult to adjust. Long et al. (2017) presented a controller for trajectory tracking under dynamic uncertainties based on active disturbance rejection control (ADRC), which facilitated the parameter tuning. First proposed by Han (2009), the core idea of ADRC is to view the system's external disturbances and internal uncertainties as “total disturbance”, estimate the real-time value of the total disturbance by an extended state observer, and finally compensate for it through feedback to achieve satisfying control performance. Moreover, Gao (2003) proposed a linear version of ADRC (LADRC), i.e., a combination of linear extended state observer (LESO) and linear state feedback, which simplified the control structure and reduced the number of tuning parameters.

Although the LADRC technique is a powerful tool to cope with dynamic uncertainties, extra model information is necessary to further improve the control performance (Li et al., 2022; Long and Peng, 2022). In this paper, a triple-step controller with LADRC (TSC-LADRC) is designed for a LLRR to accurately assist the user in tracking a predefined gait trajectory. On the one hand, the triple-step method establishes the main framework of a model-based controller. On the other hand, the feedback control is modified based on the control conception of LADRC using a second-order error auxiliary system. Accordingly, the total disturbance will be estimated in real time by the LESO and compensated with the feedback control input. To validate the effectiveness of TSC-LADRC, simulations considering the dynamic uncertainties are carried out, and experiments with the LLRR are performed on six healthy subjects. All results show that the trajectory tracking performance under TSC-LADRC is more accurate and robust than that under TSC, especially with different external loads.

## System description

### Mechanical structure

**Figure 1:**
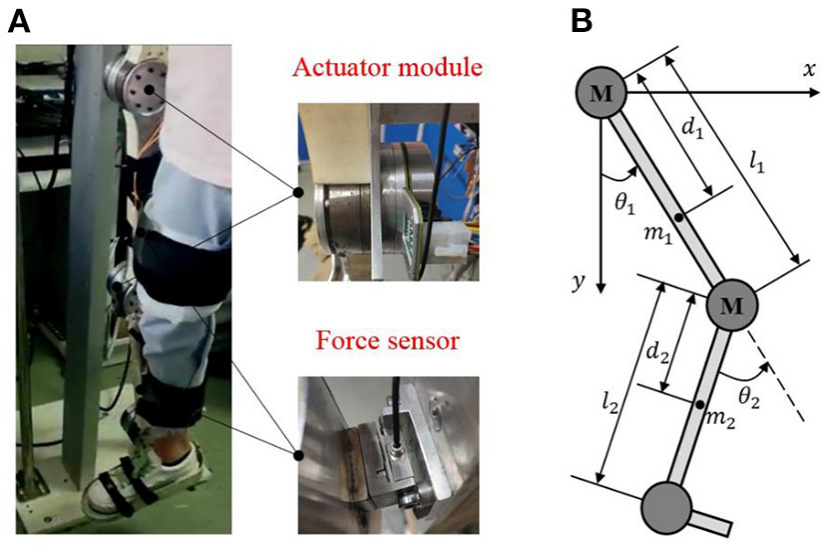
The structure of the LLRR. **(A)** is the actual prototype of the LLRR. **(B)** is the simplified two-linkage model of the LLRR.

Based on the physiological characteristics of the human's lower limb, we have developed a LLRR with three degrees of freedom, as shown in Figure 1A. The LLRR includes the hip, knee, and ankle joints, where the hip and knee joints of this LLRR are active joints driven by brushless motors (EC 90flat, Maxon, Switzerland) to assist the movement of the wearer's lower limb in the sagittal plane. The linkage is mainly made of lightweight aluminum and nylon materials through machining and three-dimensional printing. Besides, both the thigh and the shank parts are designed as a two-segment mosaic structure that can adapt to subjects of different heights. The wearer's lower limb is fixed to the exoskeleton by Velcro. And the fixed points in the limb are equipped with force sensors (FSSM-500 N, Forsentek, China), which can measure the human-robot interaction forces.

Since the exoskeleton and the wearer perform motions in a shared workspace, the designed exoskeleton must be safe. According to the ranges of motion for the lower limb exoskeleton (Veneman et al., 2007), once the program detects that the joint angle or speed is out of the normal range, the control system will immediately stop driving the motor. In addition, an emergency shutdown button is set to allow the operator to turn off the motor in time. Mechanical limit plays the ultimate role in protection. Please refer to our previous work for more details (Zhou et al., 2021).

### Dynamics model

As shown in Figure 1B, the exoskeleton can be simplified to a two-link model in the sagittal plane. Considering the external disturbances, joint friction torques as well as the uncertain model parameters, the dynamics of the LLRR can be modeled by the Euler-Lagrange method as follows:

(1) $$\begin{array}{l} \hat{M}(\theta )\ddot{\theta }+\hat{C}(\theta ,\dot{\theta })\dot{\theta }+\hat{G}(\theta )=\tau -T \end{array}$$

(2) $$\begin{array}{l} T=\tau_{HR}+f(\dot{\theta })+M(\theta )\ddot{\theta }+C(\theta ,\dot{\theta })\dot{\theta }+G(\theta ) \end{array}$$

Where $\theta =\left[\theta_{1};\theta_{2}\right]\in {\mathbb{R}}^{2\;\times \;1}$, $\dot{\theta }\in {\mathbb{R}}^{2\;\times \;1}$ and $\ddot{\theta }\in {\mathbb{R}}^{2\;\times \;1}$ are joint angle, velocity and acceleration vectors, respectively; $\tau =\left[\tau_{1};\tau_{2}\right]\in {\mathbb{R}}^{2\;\times \;1}$ are the control torques; $\text{f}\left(\dot{\theta }\right)\in {\mathbb{R}}^{2\;\times \;1}$ and ${\tau_{HR}\in \mathbb{R}}^{2\;\times \;1}$ are joint friction torques and human-robot interaction torques; $\hat{\text{M}}\left(\theta \right)\in {\mathbb{R}}^{2\;\times \;2}$, $\hat{\text{C}}\left(\theta ,\dot{\theta }\right)\in {\mathbb{R}}^{2\;\times \;2}$ and $\hat{\text{G}}\left(\theta \right)\in {\mathbb{R}}^{2\;\times \;1}$ are the nominal inertia matrix, the nominal centripetal and Coriolis matrix, and the nominal gravitational vector, respectively; **M**(***θ***) ∈ ℝ^2 × 2^, $\text{C}\left(\theta ,\dot{\theta }\right)\in {\mathbb{R}}^{2\;\times \;2}$ and **G**(***θ***) ∈ ℝ^2 × 1^ are the corresponding model uncertainties between nominal dynamics and actual dynamics; **T** ∈ ℝ^2 × 1^ is defined as the total disturbances including the structural and non-structural uncertainties.

The nominal dynamics matrixes are expressed in detail as:


(3) $$\{\begin{array}{c} \hat{M}(\theta )=\left[\begin{array}{cc} {\hat{M}}_{11}(\theta ) & {\hat{M}}_{12}(\theta )\\ {\hat{M}}_{21}(\theta ) & {\hat{M}}_{22}(\theta ) \end{array}\right]\\ {\hat{M}}_{11}(\theta )={\hat{m}}_{1}d_{1}^{2}+{\hat{m}}_{2}l_{1}^{2}+{\hat{m}}_{2}d_{2}^{2}+2{\hat{m}}_{2}l_{1}d_{2}\cos(\theta_{2})+{\hat{I}}_{1}+{\hat{I}}_{2}\\ {\hat{M}}_{12}(\theta )={\hat{m}}_{2}d_{2}^{2}+{\hat{m}}_{2}l_{1}d_{2}\cos(\theta_{2})+{\hat{I}}_{2}\\ {\hat{M}}_{21}(\theta )={\hat{M}}_{12}(\theta )\\ {\hat{M}}_{22}(\theta )={\hat{m}}_{2}d_{2}^{2}+{\hat{I}}_{2} \end{array}\;$$

(4) $$\{\begin{array}{c} \hat{C}(\theta ,\dot{\theta })=\left[\begin{array}{cc} {\hat{C}}_{11}(\theta ,\dot{\theta }) & {\hat{C}}_{12}(\theta ,\dot{\theta })\\ {\hat{C}}_{21}(\theta ,\dot{\theta }) & {\hat{C}}_{22}(\theta ,\dot{\theta }) \end{array}\right]\\ {\hat{C}}_{11}(\theta ,\dot{\theta })=-2{\hat{m}}_{2}l_{1}d_{2}\sin(\theta_{2}){\dot{\theta }}_{2}\\ {\hat{C}}_{12}(\theta ,\dot{\theta })=-{\hat{m}}_{2}l_{1}d_{2}\sin(\theta_{2}){\dot{\theta }}_{2}\\ {\hat{C}}_{21}(\theta ,\dot{\theta })={\hat{m}}_{2}l_{1}d_{2}\sin(\theta_{2}){\dot{\theta }}_{1}\\ {\hat{C}}_{22}(\theta ,\dot{\theta })=0 \end{array}$$

(5) $$\begin{array}{l} \hat{G}\left(\theta \right)=\left[\begin{array}{c} \left({\hat{m}}_{1}gd_{1}+{\hat{m}}_{2}gl_{1}\right)\sin\theta_{1}+{\hat{m}}_{2}gd_{2}\sin\left(\theta_{1}+\theta_{2}\right)\\ {\hat{m}}_{2}gd_{2}\sin\left(\theta_{1}+\theta_{2}\right) \end{array}\right] \end{array}$$

Where ${\hat{m}}_{1}$ and ${\hat{m}}_{2}$ are the mass of the thigh part and the calf part; *l*_1_ and *l*_2_ are the length of the thigh part and the calf part; *d*_1_ and *d*_2_ are the distances from the center of mass to the center of rotation; $\hat{I}$_1_ and $\hat{I}$_2_ are the moments of inertia of the thigh part and the calf part.

## Triple-step controller with LADRC

**Figure 2:**
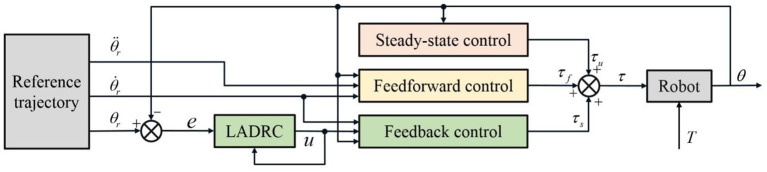
Control framework of the triple-step controller with LADRC.

In this section, a position controller for LLRR is proposed by combining the triple-step method with LADRC, in order to ensure that the robot follows the reference gait trajectory with high accuracy. Designed by the triple-step method, the control framework of the position controller is shown in Figure 2. The core concept is to divide the design process of a non-linear controller into three steps: steady-state control is to compensate for the effect of gravity, which can improve the steady-state performance of the system; feedforward control takes the change of reference dynamics into account, so as to improve the system response speed; feedback control is designed through LADRC using a second-order error auxiliary system, to reduce the influence of dynamic uncertainties during trajectory tracking.

### Steady-state control

By assigning zero to $\dot{\theta }$, $\ddot{\theta }$ and **T**, and replacing ***τ*** with ***τ***_**s**_ in (1), we can obtain the control input defined by steady-state control:

(6) $$\begin{array}{l} \tau_{s}=\hat{G}\left(\theta \right) \end{array}$$

### Feedforward control

For a complex system with non-linear and time-varying characteristics, steady-state control alone cannot achieve good control performance. Hence, a feedforward control input is designed to improve the system response speed by considering the variation of the reference dynamics.

By defining $\ddot{\theta }={\ddot{\theta }}_{r}$, $\dot{\theta }={\dot{\theta }}_{r}$, ***τ*** = ***τ***_*s*_ + ***τ***_*f*_ and assigning zero to **T** in (1), the control input defined by the reference-dynamics-based feedforward control can be obtained:

(7) $$\begin{array}{l} \tau_{f}=\hat{M}\left(\theta \right){\ddot{\theta }}_{r}+\hat{C}\left(\theta ,{\dot{\theta }}_{r}\right){\dot{\theta }}_{r} \end{array}$$

where ${\dot{\theta }}_{r}$, ${\ddot{\theta }}_{r}\in {\mathbb{R}}^{2\;\times \;1}$ are the derivative and the second derivative of the reference joint angle ***θ***_*r*_.

### Feedback control

As for the dynamics equation of the non-linear exoskeleton in (1), the uncertainties such as external disturbances and the change of structural parameters will degrade the performance of the controller. Therefore, it is significant to design a feedback control input to improve the accuracy and robustness of the non-linear system.

Since $\hat{\text{M}}\left(\theta \right)$ is a positive definite symmetric matrix, the dynamics equation (1) can be rewritten as:

(8) $$\begin{array}{l} \ddot{\theta }=\hat{M}{\left(\theta \right)}^{-1}\left(\tau -T\right)-\hat{M}{\left(\theta \right)}^{-1}\hat{C}\left(\theta ,\dot{\theta }\right)\dot{\theta }-\hat{M}{\left(\theta \right)}^{-1}\hat{G}\left(\theta \right) \end{array}$$

By letting ***τ*** = ***τ***_*s*_ + ***τ***_*f*_ + ***τ***_*u*_ and defining the tracking error **e=*****θ***_*r*_ − ***θ***, we can obtain:

(9) $$\begin{array}{l} \ddot{e}=\hat{M}{\left(\theta \right)}^{-1}\hat{C}\left(\theta ,\dot{\theta }\right)\dot{\theta }-\hat{M}{\left(\theta \right)}^{-1}\hat{C}\left(\theta ,{\dot{\theta }}_{r}\right){\dot{\theta }}_{r}\\ \;-\hat{M}{\left(\theta \right)}^{-1}\tau_{u}+\hat{M}{\left(\theta \right)}^{-1}T \end{array}$$

Based on the LADRC, a second-order error auxiliary system is defined:

(10) $$\begin{array}{l} \ddot{e}=\hat{M}{\left(\theta \right)}^{-1}T+\left(b-b_{0}\right)u+b_{0}u \end{array}$$

Where **e** = [*e*_1_; *e*_2_], **u** = [*u*_1_; *u*_2_], and **b** = *diag*(*b*_1_, *b*_2_) are the output, the input, the dynamic characteristics of the auxiliary system respectively, and **b**_0_ = *diag*(*b*_01_, *b*_02_) are the presetting values of the dynamic characteristics.

(11) $$\begin{array}{l} \tau_{u}=-\hat{M}\left(\theta \right)bu+\hat{C}\left(\theta ,\dot{\theta }\right)\dot{\theta }-\hat{C}\left(\theta ,{\dot{\theta }}_{r}\right){\dot{\theta }}_{r} \end{array}$$

The second-order auxiliary system constructed in (10) is studied as follows.

Defining the total disturbance as $\text{d}=\left[d_{1};d_{2}\right]=\hat{\text{M}}{\left(\theta \right)}^{-1}\text{T}+\left(\text{b}-{\text{b}}_{0}\right)\text{u}$, the auxiliary system can be rewritten as:

(12) $$\begin{array}{l} {ë}_{i}=d_{i}+b_{0i}u_{i},\;i=1,\;2 \end{array}$$

The core concept of LADRC is to estimate the real-time action value of the external disturbances and internal uncertainties, and compensate for it in the feedback to eliminate the influence of the total disturbance and thus enhance the performance of disturbance rejection. Specifically, the value of *d*_*i*_ can be estimated as ${\hat{d}}_{i}$ by LESO.

The extended state space model of (12) can be expressed as:

(13) $$\begin{array}{l} \{\begin{array}{c} {\dot{x}}_{i}=A_{i}x_{i}+B_{i}u_{i}+E_{i}d_{i}\\ e_{i}=C_{i}x_{i} \end{array} \end{array}$$

Where **x**_*i*_ = [*e*_*i*_; ė_*i*_; *d*_*i*_] is the extended state vector, ${\text{A}}_{i}=\left[\begin{array}{ccc} 0 & 1 & 0\\ 0 & 0 & 1\\ 0 & 0 & 0 \end{array}\right]$, **B**_*i*_ = [0; *b*_0*i*_; 0], **E**_*i*_ = [0; 0; 1], **C**_*i*_ = [1, 0, 0].

The corresponding continuous LESO is:

(14) $$\begin{array}{l} \{\begin{array}{c} {\dot{o}}_{i}=A_{i}o_{i}+B_{i}u_{i}+L_{i}\left(e_{i}-{ê}_{i}\right)\\ {ê}_{i}=C_{i}o_{i} \end{array} \end{array}$$

Where ${\text{o}}_{i}=\left[z_{i1};z_{i2};z_{i3}\right]=\left[{ê}_{i};{\hat{ė}}_{i};{\hat{d}}_{i}\right]$ is the state vector of the observer, and **L**_*i*_ = [β_*i*1_; β_*i*2_; β_*i*3_] is defined as $\left[3\omega_{oi};3\omega_{oi}^{2};\omega_{oi}^{3}\right]$, so that the gain vector of the observer is uniquely related to the bandwidth of the observer, i.e., ω_*oi*_. The explanation will be given in Section Stability of the LESO.

Replacing *e*_*i*_ − ė_*i*_ with **C**_*i*_(**x**_*i*_ − **o**_*i*_), the observer equation can be rewritten as:


(15) $$\{\begin{array}{c} \begin{array}{l} {\dot{o}}_{i}=A_{ei}o_{i}+\left[\begin{array}{cc} B_{i} & L_{i} \end{array}\right]u_{ci}\\ \;y_{i}=o_{i} \end{array} \end{array}$$

Where **u**_*ci*_ = [*u*_*i*_; *e*_*i*_] is the combined input of the observer, **y**_*i*_ = **o**_*i*_ is the observer output, and **A**_*ei*_ = **A**_*i*_ − **L**_*i*_**C**_*i*_.

For the second-order error auxiliary system, LESO can estimate the external and internal disturbances in real time. Therefore, the integrator in classical PID for eliminating static error under constant disturbance is no longer needed. The linear state feedback control law is further simplified to a proportional–derivative controller:

(16) $$\begin{array}{l} u_{0i}=-k_{Lpi}z_{i1}-k_{Ldi}z_{i2} \end{array}$$

Where *z*_*i*1_ and *z*_*i*2_ are states obtained from LESO; *k*_*Lpi*_ and *k*_*Ldi*_ are the gain coefficients, defined as $k_{Lpi}=\omega_{ci}^{2}$ and *k*_*Ldi*_ = 2ω_*ci*_, based on the closed-loop transfer function of the auxiliary system (12):

(17) $$\begin{array}{l} G_{i}=\frac{k_{Lpi}}{s^{2}+k_{Ldi}s+k_{Lpi}}=\frac{\omega_{ci}^{2}}{{\left(s+\omega_{ci}\right)}^{2}} \end{array}$$

In this way, the auxiliary system becomes a pure second-order system without zeros and the controller parameters are uniquely related to the controller bandwidth, i.e., ω_*ci*_.

**Remark 1:** The ways to determine the gains of the LESO and the linear error feedback control are termed as ω_*o*_-Optimization and ω_*c*_-Optimization respectively (Gao, 2003). The empirical value of the controller bandwidth ω_*ci*_ is one-third to one-fifth of the observer bandwidth ω_*oi*_. Generally as the value of bandwidth increases, the estimated error decreases and the controller performs better. When the bandwidth increases to a certain extent, the observer will introduce high-frequency noise and reduce the robustness of the system (Han et al., 2021). Since the bandwidths are significantly related to the control performance of the system, the tuning process of the controller is greatly simplified.

The control input of the error system is designed as follows to reject the estimated disturbance:

(18) $$\begin{array}{l} u_{i}=\frac{u_{0i}-z_{i3}}{b_{0i}}=\frac{-k_{Lpi}z_{i1}-k_{Ldi}z_{i2}-z_{i3}}{b_{0i}} \end{array}$$

Therefore, the final control input of feedback control is:

(19) $$\begin{array}{l} \tau_{u}=\hat{M}\left(\theta \right)bb_{0}^{-1}\left(K_{Lp}Z_{1}+K_{Ld}Z_{2}+Z_{3}\right)\\ \;+\hat{C}\left(\theta ,\dot{\theta }\right)\dot{\theta }-\hat{C}\left(\theta ,{\dot{\theta }}_{r}\right){\dot{\theta }}_{r} \end{array}$$

where **b** = *diag*(*b*_1_, *b*_2_), **b**_0_ = *diag*(*b*_01_, *b*_02_), **K**_*Lp*_ = *diag*(*k*_*Lp*1_, *k*_*Lp*2_), **K**_*Ld*_ = *diag*(*k*_*Ld*1_, *k*_*Ld*2_), **Z**_1_ = [*z*_11_; *z*_21_] **Z**_2_ = [*z*_12_; *z*_22_], **Z**_3_ = [*z*_13_; *z*_23_].

Sum up (6), (7), and (19), and the final control law of the triple-step controller with LADRC is obtained as:

(20) $$\begin{array}{l} \tau =\tau_{s}+\tau_{f}+\tau_{u}=\hat{M}\left(\theta \right)bb_{0}^{-1}\left(K_{Lp}Z_{1}+K_{Ld}Z_{2}+Z_{3}\right)\\ \;+\hat{M}\left(\theta \right){\ddot{\theta }}_{r}+\hat{C}\left(\theta ,\dot{\theta }\right)\dot{\theta }+\hat{G}\left(\theta \right) \end{array}$$

### Stability analysis

Assuming the derivative of the total disturbance ḋ_*i*_ is bounded, the closed-loop system (1) can be bounded-input-bounded-output stable under the triple-step controller with LADRC.

#### Stability of the LESO

Defining the estimated error of the observer as

(21) $$\begin{array}{l} e_{i}^{*}=x_{i}-o_{i}=\left[e_{i1}^{*};e_{i2}^{*};e_{i3}^{*}\right] \end{array}$$

and subtracting (13) and (14), we can obtain the error equation of LESO:

(22) $$\begin{array}{l} {\dot{e}}_{i}^{*}=A_{ei}e_{i}^{*}+E_{i}{ḋ}_{i} \end{array}$$

where

(23) $$\begin{array}{l} A_{ei}=\left[\begin{array}{ccc} -\beta_{i1} & 1 & 0\\ -\beta_{i2} & 0 & 1\\ -\beta_{i3} & 0 & 0 \end{array}\right] \end{array}$$

The characteristic polynomial of **A**_*ei*_ is

(24) $$\begin{array}{l} \lambda \left(s\right)=s^{3}+\beta_{i1}s^{2}+\beta_{i2}s+\beta_{i3} \end{array}$$

By the way of ω_*o*_-Optimization, β_*i*1_ = 3ω_*oi*_, $\beta_{i2}=3\omega_{oi}^{2}$, $\beta_{i3}=\omega_{oi}^{3}$, and thus all the roots of λ(*s*) are in the left part of the complex plane. Based on this, the LESO is obviously bounded-input-bounded-output stable because $\dot{\text{d}}$_*i*_ is bounded (Qing et al., 2007).

#### Stability analysis of the triple-step controller with LADRC

According to the second-order error auxiliary system defined by (10) and the control input defined by (18), we can obtain:

(25) $$\begin{array}{l} \{\begin{array}{c} \ddot{e}=d+b_{0}u\\ u=-b_{0}^{-1}\left(K_{Lp}Z_{1}+K_{Ld}Z_{2}+Z_{3}\right) \end{array} \end{array}$$

which can be simplified as:

(26) $$\begin{array}{l} \ddot{e}+K_{Lp}Z_{1}+K_{Ld}Z_{2}+Z_{3}=d \end{array}$$

Combining (21) with (26), the dynamics equation of the tracking error can be obtained:

(27) $$\begin{array}{l} \ddot{e}+K_{Lp}e+K_{Ld}\dot{e}=K_{Lp}E_{1}^{*}+K_{Ld}E_{2}^{*}+E_{3}^{*} \end{array}$$

where ${\text{E}}_{1}^{*}=\left[e_{11}^{*};e_{21}^{*}\right]$, ${\text{E}}_{2}^{*}=\left[e_{12}^{*};e_{22}^{*}\right]$, ${\text{E}}_{3}^{*}=\left[e_{13}^{*};e_{23}^{*}\;\right]$.

As stated in Section Stability of the LESO, as long as **A**_*ei*_ is a Hurwitz matrix and $\dot{\text{d}}$ is bounded, the boundness of ${\text{E}}_{1}^{*}$, ${\text{E}}_{2}^{*}$ and ${\text{E}}_{3}^{*}$ can be guaranteed. Besides, **K**_*Lp*_ and **K**_*Ld*_ are positive-definite by the way of ω_*c*_-Optimization. Therefore, according to the Routh criterion, the tracking error **e** is bounded and the system is bounded-input-bounded-output stable.

## Simulation

### Simulation setup

The uncertainties of the dynamics model, such as the uncertainty of model parameters, sensor measurement noises, external disturbances, load changes and so on, have a significant impact on the performance of a model-based control method. In this section, numerical simulations with uncertainties are carried out in Matlab (R2020b, MathWorks), to verify the excellent performance of the triple-step controller with LADRC (TSC-LADRC) compared with the original triple-step controller (TSC). The control law of TSC is expressed as (Zhou et al., 2019):

(28) $$\begin{array}{l} \tau =\hat{M}\left(\theta \right)\left(K_{p}e+K_{i}\int edt+K_{d}\dot{e}\right)+\hat{M}\left(\theta \right){\ddot{\theta }}_{r}\\ \;+\hat{C}\left(\theta ,\dot{\theta }\right)\dot{\theta }+\hat{G}\left(\theta \right)-\tau_{HR} \end{array}$$

Where **K**_*p*_, **K**_*i*_ and **K**_*d*_ denote the proportional, the integral and the derivative gain vectors.

The nominal physical parameters are set as ${\hat{m}}_{1}=2.582\;kg$, ${\hat{m}}_{2}=3.192\;kg$, *l*_1_ = 0.390 *m*, *l*_2_ = 0.464 *m*, *d*_1_ = 0.328 *m*, *d*_2_ = 0.355 *m*, and the actual mass parameters are set as $m_{1}={\hat{m}}_{1}\times 120\%$, $m_{2}={\hat{m}}_{2}\times 120\%$. Referring to Yang et al. (2019), the interaction torques are assumed to be periodic, i.e., ***τ***_*HR*_ = [2 cos (0.2π*t*); 2 sin (0.2π*t*)]. The reference angle of each joint is fitted from a healthy subject's gait data (Zhou et al., 2021). The trajectory's period is set to 10s and a simulation includes three cycles. All the simulations are conducted with a sample time 0.01s. For simplicity, the observer bandwidths of hip and knee joints are set to be the same value ω_*o*_ = 60, and the controller bandwidth is one-third of the observer bandwidth. Moreover, **b** = *diag*(10, 10),**b**_0_ = *diag*(5, 5),**K**_*p*_ = *diag*(950, 1, 020),**K**_*i*_ = *diag*(80, 90),**K**_*d*_ = *diag*(110, 130).

The interaction between the wearer and the exoskeleton can be measured by force sensors. However, it is difficult to accurately measure the disturbances exerted by the time-varying load through the force sensors. Hence, in order to demonstrate the robust control performance of TSC-LADRC against external loads, three sets of time-varying external torques with different magnitudes are applied to the system to simulate different external loads, i.e., (I) τ_*L*1_ = 5 cos(0.4π*t*), τ_*L*2_ = 5 sin(0.4π*t*); (II) τ_*L*1_ = 10 cos(0.4π*t*), τ_*L*2_ = 10 sin(0.4π*t*); and (III) τ_*L*1_ = 15 cos(0.4π*t*), τ_*L*2_ = 15 sin(0.4π*t*).

### Simulation results

**Figure 3:**
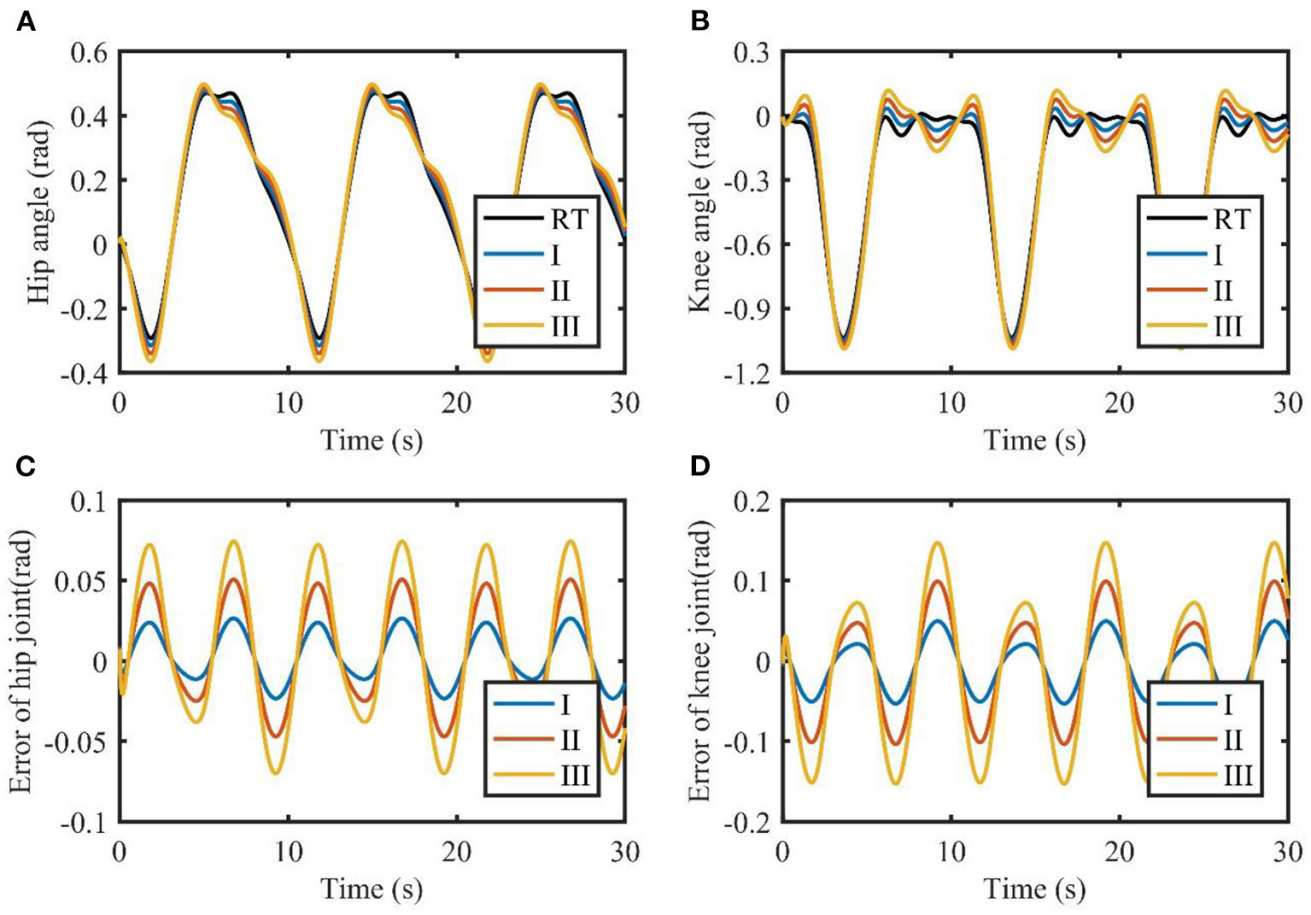
Tracking performance of TSC with different loads. **(A,B)** are the joint angles of the hip and knee. **(C,D)** are the tracking errors of the hip and knee. I, II, and III represent three cases of different loads. RT, reference trajectory.

**Figure 4:**
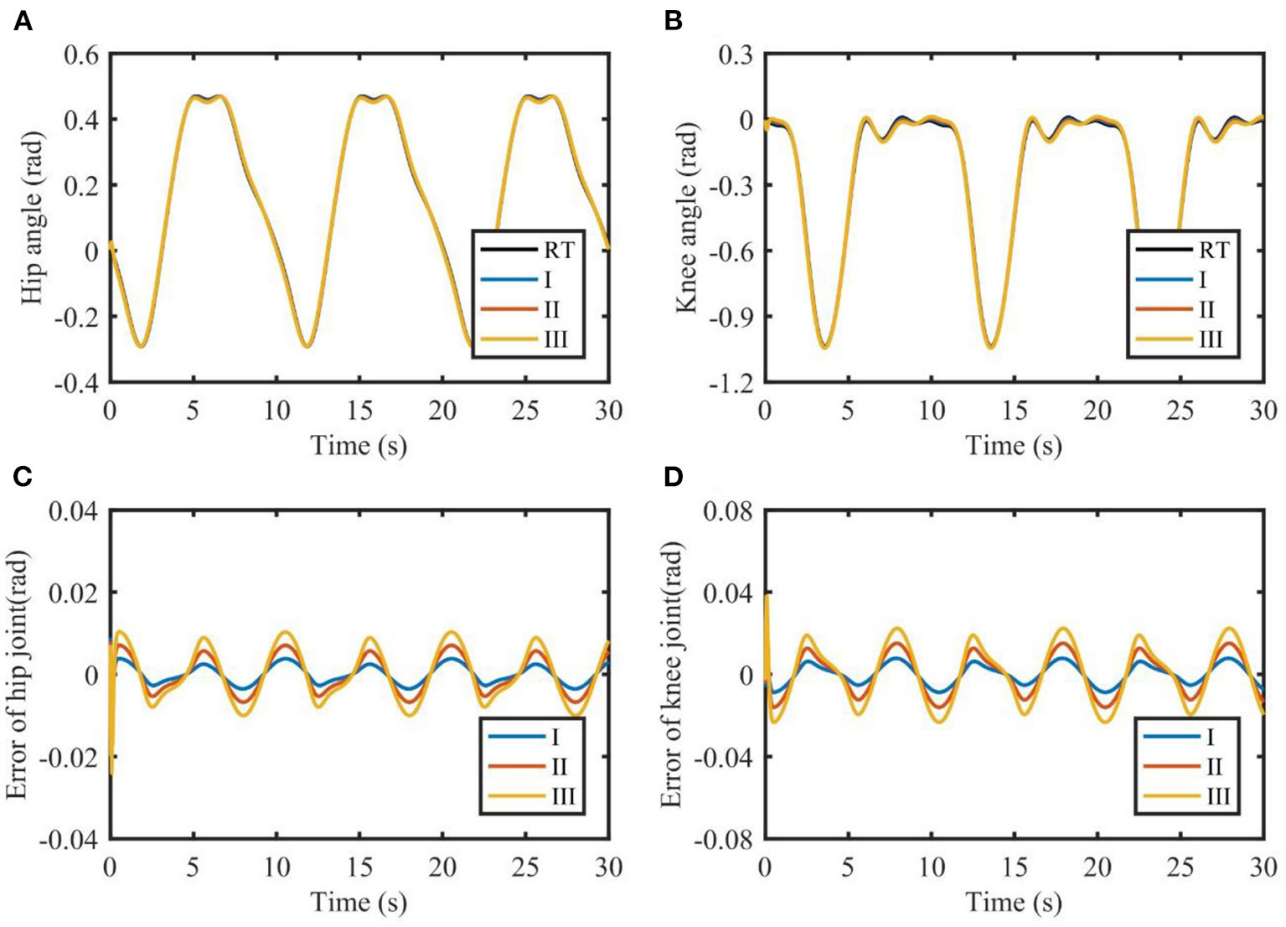
Tracking performance of TSC-LADRC with different loads. **(A,B)** are the joint angles of the hip and knee. **(C,D)** are the tracking errors of the hip and knee. I, II, and III represent three cases of different loads. RT, reference trajectory.

The results of the simulations are shown in Figures 3, 4. It can be seen from Figure 3 that TSC is able to follow the reference trajectories. However, the presence of model uncertainties makes it difficult to reduce the tracking errors. Furthermore, TSC is susceptible to the changes of the load. As the load increases, the tracking errors of TSC increase significantly, with the maximum errors of the two joints exceeding 0.05 and 0.1 rad, respectively. By contrast, the absolute value of the tracking errors shown in Figures 4C,D are almost less than 0.02 and 0.04 rad, respectively, demonstrating that the proposed TSC- LADRC is robust against different external loads and can achieve higher control accuracy.

**Figure 5:**
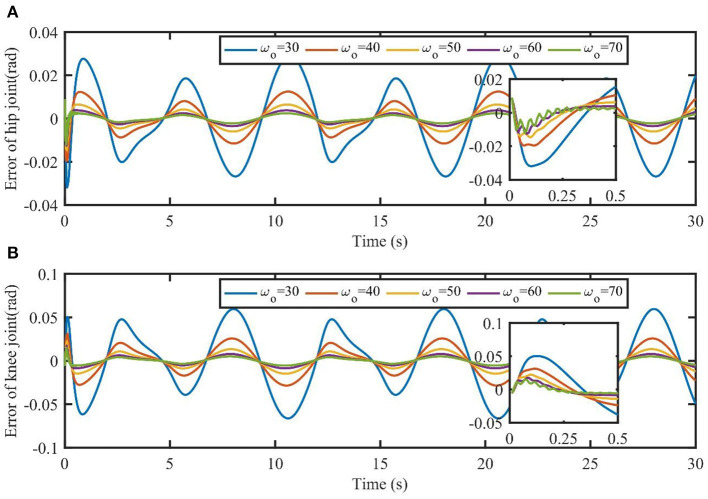
Tracking errors of TSC-LADRC under different values of ω_*o*_. The part plots are the initial response of joints' tracking errors in the time interval [0, 0.5s].

To further verify the effect of the observer bandwidth described in Remark 1, the simulation results of TSC-LADRC under different bandwidths are shown in Figure 5. External loads are fixed to τ_*L*1_ = 5 cos(0.4π*t*) and τ_*L*2_ = 5 sin(0.4π*t*). For simplicity, the observer bandwidths of hip and knee joints keep the same value ω_*o*_. From Figure 5, the larger the value of ω_*o*_, the smaller the tracking errors, which means larger observer bandwidths can enhance the control performance of TSC-LADRC. Besides, when the bandwidth increases to 70, the observer will introduce high-frequency noise and reduce the smoothness of the trajectories. These results are consistent with what we described in **Remark 1**, hence we can intuitively set the parameters by control performance.

## Experiment

### Experiment protocol

To further validate the superiority of the proposed method to the original triple-step controller, experiments were conducted on the actual exoskeleton robot, based on LADRC-TSC and TSC, respectively. Six healthy subjects were recruited to perform passive trajectory tracking experiments on the LLRR (four males and two females; age, 24.33 ± 2.56 years; height, 1.69 ± 0.07 m; weight, 59.33 ± 7.76 kg). The reference angle of each joint and the sampling time were set the same as those in the simulations. Each subject was asked to perform five experiments for each controller. The experimental operator assisted the subjects in getting familiar with the LLRR before the experiments. Besides, the ethical approval of our study was authorized by the Ethics Committee of Guangdong Work Injury Rehabilitation Center and written informed consents were signed by all subjects. The control parameters of TSC and TSC-LADRC shown in Table 1 are chosen according to the control performance by a trial-and-error method.

**Table 1:** Control parameters.

**Method**	**Parameter**	**Value**
TSC-LADRC	**b**	*diag*(0.3, 0.5)
	**b**_0_	*diag*(1, 1)
	**ω**_*o*_	*diag*(36, 36)
	**ω**_*c*_	*diag*(12, 12)
TSC	**K**_*p*_	*diag*(76.01, 126.78)
	**K**_*i*_	*diag*(91.28, 253.57)
	**K**_*d*_	*diag*(2.11, 5.87)

During the experiments, the workflow of the robotic system can be described as follows. First, the actual positions of the joints are measured using the angle encoders, which are assembled with the joint motors. Second, a data acquisition board (NI USB-6341, National Instruments, USA) transfers the angle data to a laptop computer with an Intel i5 12500H CPU (2.5 GHz) and 16 GB of RAM. Next, the computer processes the signals in LabVIEW 2018 software based on the position controller. The software generates the control input and translates it into pulse width modulation (PWN) signals. Finally, the PWN signals are transferred to the data acquisition board through a USB interface, and the board sends the signals to the motor drivers, which can supply specific voltages for the joint motors. Meanwhile, the actual positions of the joints are measured again. Therefore, the robot can be driven to follow the reference angles based on this workflow.

### Evaluation method

To evaluate the control performance of TSC-LADRC, we calculate the root mean square error (RMSE) based on the trajectory tracking errors of the hip and knee joints, respectively:

(29) $$\begin{array}{l} RMSE=\sqrt{\frac{1}{n}\sum_{k=1}^{n}e^{2}\left(k\right)} \end{array}$$

Where *e*(*k*) is the tracking error at the *k*th sampling time point, and *n* is the sample number.

Besides, we calculate the amount of energy that motors consume during trajectory tracking, based on an energy index *E* (Jiang et al., 2017):

(30) $$\begin{array}{l} E=\sum_{i=1}^{2}\int_{0}^{l}|\tau_{i}\left(t\right)|dt \end{array}$$

Where τ_*i*_ (*i* = 1, 2) is the control torque and *l* is the length of the torque signal.

The two evaluation indicators were calculated across each experiment. All the indicators are expressed in the form of mean ± standard error. And the paired *t*-test with a significance level of 0.05 was utilized to test the effect of the control algorithms statistically.

### Experiment results

**Figure 6:**
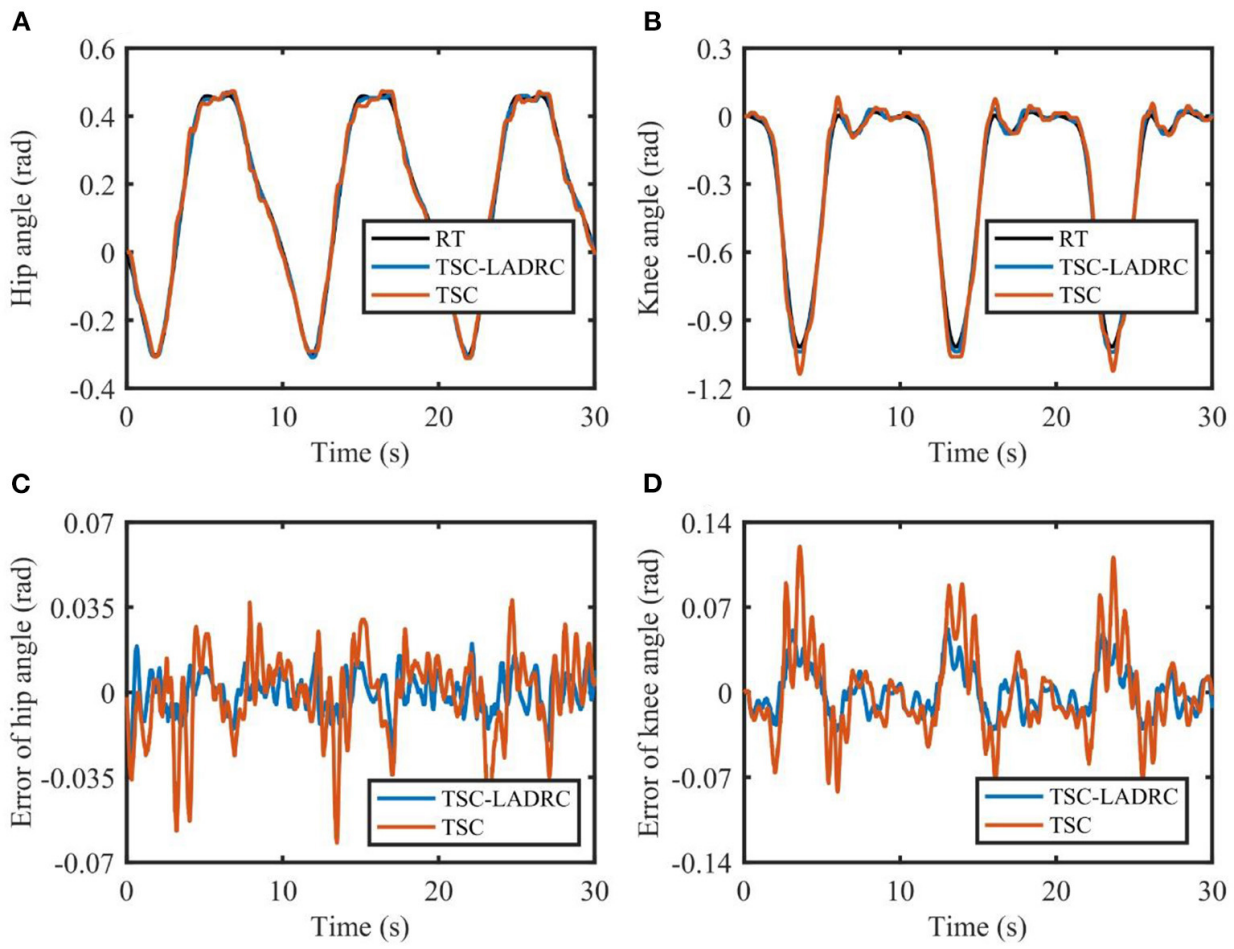
Tracking performance of the exoskeleton robot under TSC-LADRC and TSC. **(A,B)** are the joint angles of the hip and knee. **(C,D)** are the tracking errors of the hip and knee. RT, reference trajectory.

The tracking results in one experiment are shown in Figure 6. TSC can assist the LLRR in following the reference angles. However, due to the factors such as friction, sensor noise, model uncertainties, and so on, the actual trajectories based on TSC have undesirable chattering phenomena and deviate from the reference trajectories at some point. Compared with TSC, the proposed TSC-LADRC is able to realize more accurate trajectory tracking, and the problem of chattering can be reduced. From Figures 6C,D, the maximum tracking errors of TSC-LADRC are almost half smaller than those of TSC.

**Figure 7:**
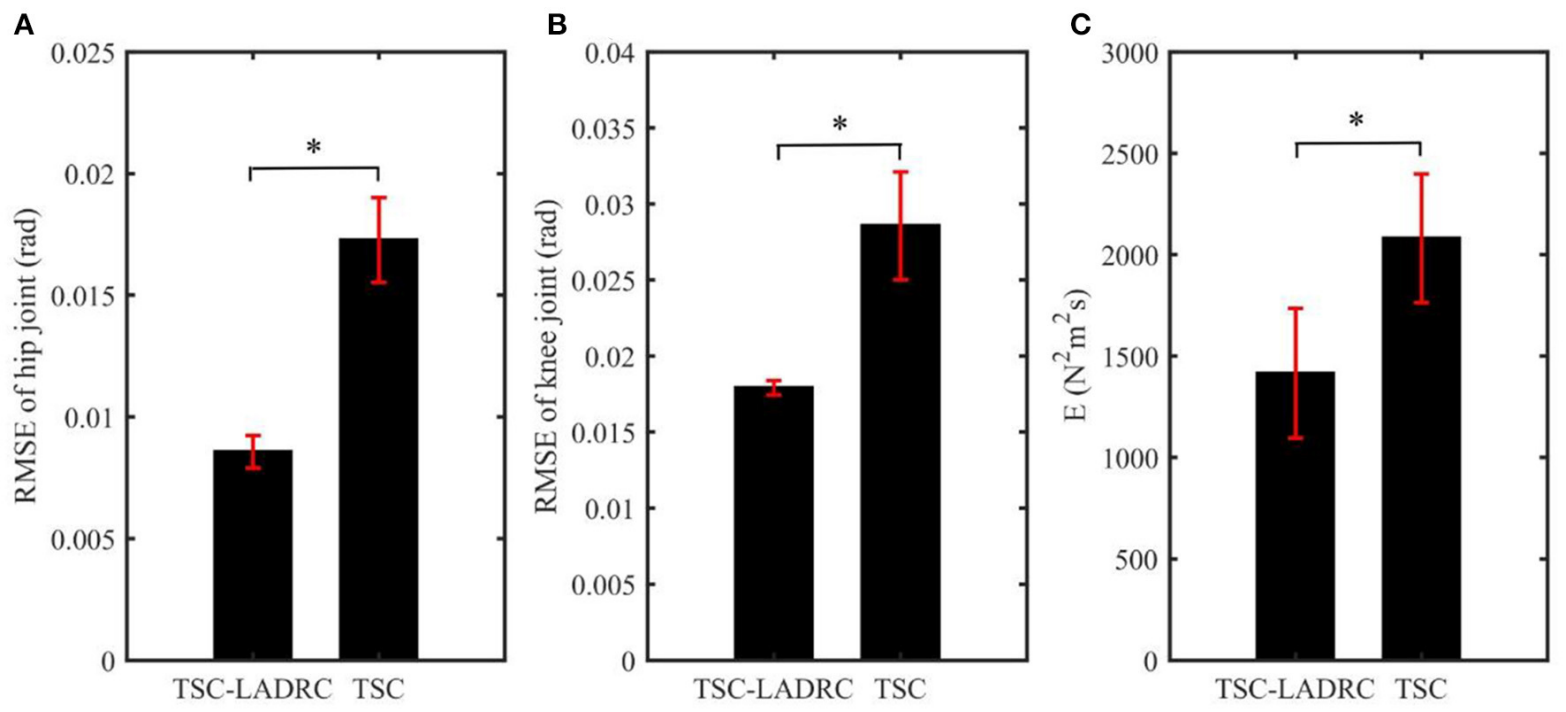
The mean values of each evaluation index in all experiments: **(A)** RMSE results of hip joint; **(B)** RMSE results of knee joint; **(C)** Energy index. The error bars indicate the standard errors. The asterisk reveals significant difference (p < 0.05).

The calculation results of RMSE are shown in Figures 7A,B. The RMSE results of the TSC- LADRC are 0.0086 ± 0.0007 and 0.0179 ± 0.0005 rad, while the RMSE results of TSC are 0.0173 ± 0.0017 and 0.0286 ± 0.0035 rad, respectively. It can be seen from Figures 7A,B that the RMSE results of the TSC-LADRC are reduced significantly by 50.29 and 37.41% respectively compared with those of TSC. Besides, the standard error of the RMSE results under TSC-LADRC is less than that under TSC, which means that the proposed controller is more robust than the TSC when coping with different loads. Hence, the TSC-LADRC can improve the accuracy and robustness of trajectory tracking for the lower limb rehabilitation robot.

The energy consumption of each controller is given in Figure 7C. We can see that the energy consumption of LADRC-TSC is less than that of TSC in all experiments. The average energy consumption of LADRC-TSC is 1,414.62 *N*^2^*m*^2^*s* and that of TSC is 2,081.00 *N*^2^*m*^2^*s*, showing a significant difference. From the above results, it can be concluded that TSC-LADRC not only realizes more accurate and robust trajectory tracking but also achieves less energy consumption than TSC.

## Discussion

Control accuracy and robustness are critical during robot-aided rehabilitation training, while LLRR is vulnerable to dynamic uncertainties due to the unexpected behavior of stroke patients, model uncertainties, and external disturbances (Yang et al., 2019, 2020). In this study, TSC-LADRC was a robust position controller that addressed the dynamic uncertainties in trajectory tracking through a simple and easy-to-apply control structure.

Our previous work has verified that TSC can guarantee control accuracy under different interaction torque levels (Zhou et al., 2019). However, the simulation results in this study revealed that the model errors and external loads would degrade the tracking performance of TSC. Compared with TSC (Zhou et al., 2019), the key feature of TSC-LADRC was to define a second-order error auxiliary system, which could estimate and reject the total uncertainties based on the LADRC (Gao, 2003). On the other hand, the parameter tuning of TSC-LADRC was more straightforward than that of TSC. Based on LADRC (Gao, 2003), TSC-LADRC had only two main parameters to be tuned, the controller bandwidth ω_*ci*_ and the observer bandwidth ω_*oi*_. Moreover, an empirical setting of ω_*ci*_ was $\frac{1}{5}\sim \frac{1}{3}\omega_{oi}$, meaning that the tuning of TSC-LADRC was further simplified. From the simulation results shown in Figure 5, the tracking errors decreased with the observer bandwidth increasing, which was consistent with the results of Long et al. (2017). Therefore, the main control parameter ω_*oi*_ of TSC-LADRC was directly related to the control performance and easy to be tuned.

Trajectory error directly reflects the tracking ability of the position controller. Compared with TSC, the decrease in RMSE values of TSC-LADRC demonstrated that the tracking ability of TSC-LADRC improved significantly, which could be explained by the reason that the total disturbances of the robotic system were estimated and compensated by the LADRC-based feedback control. It is worth mentioning that, the RMSE values of TSC-LADRC were smaller than the RMSE results of Huang et al. (2022), and the tracking errors shown in Figures 6C,D were under the average errors of Zhang et al. (2020). This means that, compared with the DO-based controller (Huang et al., 2022) and RBFNN-based controller (Zhang et al., 2020), TSC-LADRC not only facilitates the parameter tuning, but can also address the dynamic uncertainties and improve the tracking accuracy. Moreover, compared with TSC, the decrease in *E* value of TSC-LADRC demonstrated that the energy consumption efficiency of the controller improved significantly (Jiang et al., 2017). We attributed this phenomenon to the fact that, by combining LADRC with TSC, small and bounded tracking errors were guaranteed, which could also lead to a smaller feedback gain in the control law. For the rehabilitation robot system, the energy reduction is beneficial to improving the portability of the exoskeleton design (Ferris et al., 2007).

In future work, experiments will be carried out on patients with motor dysfunction to further verify the clinical effectiveness of TSC-LADRC. Moreover, the LLRR will be combined with treadmill and the motor performance of the wearer's non-paretic limb will be assessed in real time. Based on this real-time assessment, we will focus on the adaptation law of the observer bandwidth to improve patients' gait symmetry and promote their active effort (Wolbrecht et al., 2008; Zhong et al., 2022).

## Conclusion

In this study, a triple-step controller with LADRC was proposed for a LLRR to improve gait tracking performance. Under the design framework of the triple-step method, LADRC was incorporated into the feedback control to improve the accuracy and robustness against dynamic uncertainties. Results of numerical simulations and experiments showed that TSC-LADRC could achieve better control performance than TSC. Moreover, our proposed controller facilitated the tuning of control parameters. Therefore, it has the potential to be an easy-to-implement position controller for LLRRs to achieve promising performance, and can be extended to other rehabilitation robots.

## Data availability statement

The raw data supporting the conclusions of this article will be made available by the authors, without undue reservation.

## Ethics statement

The studies involving human participants were reviewed and approved by Ethics Committee of Guangdong Work Injury Rehabilitation Center. The patients/participants provided their written informed consent to participate in this study.

## Author contributions

JZ and RS contributed conception of the control algorithm. HP designed and performed the simulations, experiments, and wrote the first draft of the manuscript. All authors contributed to the manuscript revision and approved the submitted version.

## Funding

This research was supported by the National Key Research and Development Program of China (Grant No. 2022YFE0201900), the Shenzhen Science and Technology Research Program (Grant No. SGDX20210823103405040), the Guangdong Science and Technology Plan Project (Grant No. 2020B1212060077) and the Natural Science Foundation of Guangdong Province (Grant No. 2020A1515010735).

## Conflict of interest

The authors declare that the research was conducted in the absence of any commercial or financial relationships that could be construed as a potential conflict of interest.

## Publisher's note

All claims expressed in this article are solely those of the authors and do not necessarily represent those of their affiliated organizations, or those of the publisher, the editors and the reviewers. Any product that may be evaluated in this article, or claim that may be made by its manufacturer, is not guaranteed or endorsed by the publisher.
